# ﻿Two new and one new morph record of *Calycina* (Pezizellaceae, Helotiales) species from Southwestern China

**DOI:** 10.3897/mycokeys.122.158883

**Published:** 2025-09-04

**Authors:** Le Luo, Kandawatte Wedaralalage Thilini Chethana, Qi Zhao, Vinodhini Thiyagaraja, Kitiphong Khongphinitbunjong, Fatimah Al-Otibi, Kevin D. Hyde

**Affiliations:** 1 State Key Laboratory of Phytochemistry and Natural Medicines, Kunming Institute of Botany, Chinese Academy of Sciences, Kunming 650201, Yunnan, China; 2 Center of Excellence in Fungal Research, Mae Fah Luang University, Chiang Rai 57100, Thailand; 3 School of Science, Mae Fah Luang University, Chiang Rai 57100, Thailand; 4 Guiyang Institute of Humanities and Technology, Guiyang 550025, China; 5 Department of Botany and Microbiology, College of Science, King Saud University, P.O. Box 22452, Riyadh 11495, Saudi Arabia

**Keywords:** Asexual morph, Leotiomycetes, morphology, phylogeny, sexual morph

## Abstract

*Calycina* (Pezizellaceae, Helotiales) is a heterogeneous group, and the taxonomic classification is frequently revised due to unclear phylogenetic relationships. To date, a total of 35 species in this genus have been documented, with six recorded in China, including four species that are endemic to the country. *Calycina* species are reported in their sexual and asexual morphs, and the phylogenetic placements of several species remain unresolved due to the lack of molecular data. During a study on the fungal diversity of southwestern China, six specimens were collected and analyzed using both morphological characterizations and multi-gene phylogenetic analyses (LSU and ITS). The results revealed two new species, *C. botanica* and *C. xishuangbanica*, and the first sexual report of *C. brevipes*, with detailed morphological descriptions and phylogenetic trees.

## ﻿Introduction

*Calycina* was originally proposed as an infrageneric taxon of *Peziza*, including four species: *Peziza firma*, *P. herbarium*, *P. citrina*, and *P. bolaris* (Nees von Esenbeck 1817). Later, Gray (1821) raised *Calycina* to generic rank and retained *P. firma* and *P. herbarium*, while adding three new species: *P. subsimilis*, *P. succosa*, and *P. sulphurea*. Seaver (1934) initially designated *Peziza firma* as the type of *Calycina*, a species which was treated by Gray (1821). However, Seaver (1934) typification was not accepted as that study failed to select one of the four eligible species. Consequently, *C. herbarum* (= *Peziza herbarium*) was later designated as the type (Dumont 1972).

The familial placement of *Calycina* has been debated over the years. Han et al. (2014) placed *Calycina* within Hyaloscyphaceae, as *C. herbarum* was found to cluster with *Calycellina populina* in a strongly supported clade based on the phylogenetic analyses of ITS, LSU, *rpb2*, and mtSSU sequences. Morphologically, both genera possess hairs that are much shorter and indistinct from the extant genera of Hyaloscyphaceae (Gray 1821; Dumont 1972; Lowen and Dumont 1984). Therefore, Baral and Rämä (2015) proposed combining *Calycellina populina* under *Calycina populina* based on the phylogenetic analysis of ITS sequence data. Later, Jaklitsch et al. (2016) accepted *Calycina* under Pezizellaceae (Helotiales, Leotiomycetes) based on the phylogenetic framework established by Han et al. (2014). The genus was distinguished from Hyaloscyphaceae*sensu stricto* by having frequently observed vacuolar bodies and the occurrence of a Chalara-type anamorph, a distinction that was also accepted in later studies (Wijayawardene et al. 2017, 2020, 2022; Hyde et al. 2024). *Calycina* was considered polyphyletic in the recent phylogenetic analyses conducted by Wu and Diao (2023), similar to previous studies (Baral and Rämä 2015; Guatimosim et al. 2016; Suija and Motiejūnaité 2016; Friggens et al. 2017; Crous et al. 2019; Karunarathna et al. 2021; Mitchell et al. 2022). There are 35 species in *Calycina* recorded in Species Fungorum (2025); however, molecular data are unavailable for only 18 species.

Species of *Calycina*, particularly those with Chalara-like anamorphs, are primarily reported as saprobes on plant materials and fucoid alga (Baral and Rämä 2015; Quijada and Beltrán-Tejera 2017), endophytes in living plants (Crous et al. 2019), or as lichenicolous taxa (Suija and Motiejūnaitė 2017). They are reported in both sexual and asexual morphs. The sexual morph is characterized by bright-coloured apothecia, gelatinized ectal excipulum, filiform paraphyses, cylindrical asci, and hyaline, fusoid to ellipsoid ascospores (Baral and Rämä 2015; Suija and Motiejūnaitė 2017; Lestari and Chethana 2022). The asexual morph is characterized by the presence or absence of stroma. The conidiophores are macronematous or micronematous, scattered or aggregated from the basal stroma, erect, simple, or rarely branched, subcylindrical, and range in color from pale brown to very dark brown. They are smooth or verrucose, septate, consisting of a basal cell or septate stalk and a terminal phialide, with or without percurrent proliferations. The conidiogenous cells are ampulliform, lageniform, obclavate, ellipsoidal, urceolate, or subcylindrical, ranging from pale brown to dark brown, with a venter and a collarette. The transition from venter to collarette can be gradual, abrupt, or barely perceptible. The conidia are holoblastic, cylindrical, straight, hyaline, aseptate, smooth, with rounded or truncate ends, often featuring a basal marginal frill or, in rare cases, fringes of wall material slightly rounded at the apex, and are extruded in short chains (Wu and Diao 2023). Only three species, *Calycina lactea*, *C. parilis* and *C. vulgaris*, were known from both anamorphs and teleomorphs (Wu and Diao 2023).

Further investigation is essential to refine the generic concept and to resolve phylogenetic relationships. During studies on Leotiomycetes, six collections of *Calycina* were obtained. Morphological and phylogenetic analyses based on the combined LSU and ITS sequences identified these collections as three species: two new species (*Calycina botanica* and *C. xishuangbanica*), and the sexual morph of a known asexual species, *C. brevipes*.

## ﻿Material and methods

### ﻿Collection and morphological examination

We collected six specimens of decaying wood and dead leaves from Yunnan Province, including the cities of Kunming, Xishuangbanna, and Pu’er in southwestern China. All samples were obtained from highly humid, natural broadleaf forests and protected areas with minimal human disturbance. The samples were dried using a dehydrator set at 25–30 °C. After studying the morphology of the specimens and getting their genomic DNA, they were deposited at the Cryptogamic Herbarium of the Kunming Institute of Botany, Chinese Academy of Sciences (**KUN-HKAS**). Facesoffungi and Index Fungorum numbers were obtained as in Jayasiri et al. (2015) and Index Fungorum (2025), respectively. The morphological descriptions were submitted to the Greater Mekong Subregion database (Chaiwan et al. 2021). The dried specimens were examined with a stereomicroscope (C-PSN, Nikon, Japan) and were captured with a digital camera (Canon EOS 70D, Japan) connected to the stereomicroscope. Free-hand sections of the dried specimens were mounted in a drop of water for observing microscopic characteristics, such as apothecia, exciple, paraphyses, asci and ascospores, using a Nikon compound microscope (Nikon, Japan) equipped with a DS-Ri2 camera. In addition, the sections were pretreated with Melzer’s reagent for the Iodine test (MLZ) (Tochihara and Hosoya 2022). Microstructures were measured using the Tarosoft(R) Image Frame Work program v.0.97 (Tarosoft, Thailand). The obtained measurements were presented in the format of (a–) b–c(–d), where ‘a’ represented the minimum value, ‘d’ represented the maximum value, and the range ‘b–c’ reflected the 90% confidence interval. Images used for figures were processed with Adobe Photoshop CS6 Extended version 13.0 × 64 (Adobe Systems, USA).

### ﻿DNA extraction, PCR amplifications and sequencing

Genomic DNA was extracted from the dried apothecia using a TSP101 DNA extraction kit (TSINGKE, China). Following the latest studies (Su et al. 2023; Luo et al. 2024), LSU and ITS regions were used for PCR amplification with the primers LR0R/LR5 (Vilgalys and Hester 1990) and ITS1F/ITS4 (White et al. 1990; Gardes and Bruns 1993), respectively. The total volume of a PCR reaction was 25 μL, comprising 12.5 μL 2×PCR G013 Taq MasterMix with Dye (Applied Biological Materials, Canada), 1 μL of each primer (10 μM), 2 μL genomic DNA, and 8.5 μL of sterilized, distilled water. Amplifications of LSU and ITS regions were conducted under the following conditions: pre-denaturation at 95 °C for 5 min, followed by 35 cycles of denaturation at 95 °C for 20 sec, annealing at 56 °C (LSU)/53 °C (ITS) for 10 sec, elongation at 72 °C for 20 sec, and a final elongation at 72 °C for 7 min. Gel electrophoresis with 1% TAE and TSJ003 GoldView nucleic acid dye (TSINGKE, China) was used to confirm the obtained PCR products. Finally, the PCR products were sequenced at the Tsingke Biotechnology Co., Ltd., Kunming, China. Newly produced sequences were deposited in the GenBank and the accession numbers were given in Table 1.

**Table 1.** Taxa included in the phylogenetic analyses and the GenBank accession numbers of LSU and ITS sequences.

Species	Strain	Gene accession No.		Reference
		ITS	LSU	
*Austropezia samuelsii*	PDD:64271	MH578480	–	Unpublished
*Bisporella pallescens*	DMS-10078235^T^	MW203182	–	Unpublished
*Bisporella pallescens*	DMS-10075832	MW203180	–	Unpublished
*Bisporella subpallida*	GM20160214	KY462818	–	Lestari and Chethana (2022)
*Bloxamia descedens*	16029	ON993899	OP173629	Li et al. (2024)
*Bloxamia descedens*	8140b	ON993900	–	Wu and Diao (2023)
*Bloxamia elegans*	352173^(T)^	ON993901	–	Wu and Diao (2023)
*Bloxamia elegans*	17130c	ON993903	–	Wu and Diao (2023)
*Bloxamia elongata*	1041f	ON993904	–	Wu and Diao (2023)
*Bloxamia elongata*	352174^(T)^	ON993905	OP173630	Wu and Diao (2023)
*Bloxamia truncata*	8259b	ON993907	OP173631	Wu and Diao (2023)
*Bloxamiella cyatheicola*	VIC 42563^(T)^	NR153617	NG058691	Guatimosim et al. (2016)
*Bloxamiella cyatheicola*	VIC:42460	KU597792	KU597759	Guatimosim et al. (2016)
*Calycellina fagina*	TK7178	MT231710	–	Kosonen et al. (2021)
*Calycellina fagina*	SBRH925	OL752703	OM218631	Unpublished
*Calycellina lachnobrachya*	190571597	OR872184	–	Kõljalg et al. (2024)
*Calycellina lachnobrachyoides*	KUS-F52576	JN033424	–	Han et al. (2014)
*Calycellina lachnobrachyoides*	KUS-F52183	JN033412	–	Han et al. (2014)
*Calycellina populina*	CBS:247.62	MH858147	MH869739	Vu et al. (2019)
*Calycellina populina*	KACC45615	JN033382	JN086685	Han et al. (2014)
*Calycellina punctata*	Cantrell GA18	U57494	–	Unpublished
*Calycellina ulmarie*	K(M):200003	MZ159550	–	Lestari and Chethana (2022)
*Calycina afnis*	16142a	ON993909	OP173632	Wu and Diao (2023)
*Calycina afnis*	16147a	ON993912	OP173633	Wu and Diao (2023)
*Calycina alstrupii*	Pz167	KY305096	KY305098	Suija and Motie (2017)
*Calycina alstrupii*	10761^(T)^	NR154846	NG068538	Suija and Motie (2017)
***Calycina botanica***	**HKAS 139496**	**PV138605**	**PV156742**	This study
***Calycina botanica***	**HKAS 139497^(T)^**	**PV138606**	**PV156743**	This study
*Calycina brevipes*	8280	ON993924	OP173639	Wu and Diao (2023)
*Calycina brevipes*	8302	ON993925	OP173640	Wu and Diao (2023)
***Calycina brevipes***	**HKAS 139498**	**PV138609**	**PV156746**	This study
***Calycina brevipes***	**HKAS 139499**	**PV138610**	**PV156747**	This study
*Calycina citrina*	FH LQH-9a	MW203183	MW115642	Wu and Diao (2023)
*Calycina citrina*	Au96-119	PP701694	–	Unpublished
*Calycina claroflava*	F132983	KC412006	–	Baral et al. (2013)
*Calycina cortegadensis*	MSS906	MN017444	MN017503	Unpublished
*Calycina crassipes*	CBS82971	ON993926	–	Wu and Diao (2023)
*Calycina discreta*	voucher 7511	JF908571	–	Osmundson et al. (2013)
*Calycina dualis*	ICMP:14950	EF029209	–	Unpublished
*Calycina ellisii*	M01452:264	LR875927	–	Unpublished
*Calycina eucalypticola*	NN043033	ON993929	–	Wu and Diao (2023)
*Calycina eucalypticola*	NN043157	ON993928	–	Wu and Diao (2023)
*Calycina eucalypticola*	CPC 36078^(T)^	NR_182461	NG_148969	Unpublished
*Calycina fungorum*	CBS94272	ON993930	–	Wu and Diao (2023)
*Calycina fungorum*	CBS40581	ON993964	–	Wu and Diao (2023)
*Calycina herbarum*	KUS-F52362	JN033407	JN086710	Han et al. (2014)
*Calycina herbarum*	KUS-F51458	JN033390	JN086693	Han et al. (2014)
*Calycina lactea*	iNAT:18000156	MZ209003	–	Lestari and Chethana (2022)
*Calycina lactea*	HB7224	KC412007	–	Baral et al. (2013)
*Calycina languida*	F116599	KC412002	–	Baral et al. (2013)
*Calycina languida*	F116600	KC412003	–	Baral et al. (2013)
*Calycina marina*	TROM F26093	KT185677	KT185670	Baral et al. (2015)
*Calycina marina*	TROM:F26101	KT185674	–	Baral et al. (2015)
*Calycina montana*	MFLU 22-0055	ON156524	ON156523	Lestari and Chethana (2022)
*Calycina montana*	MFLU 22-0056	ON176689	ON325438	Lestari and Chethana (2022)
*Calycina montana*	HMAS 275566^(T)^	NR153627	–	Mitchell et al. (2022)
*Calycina oxenbolliae*	NN050633	ON993940	–	Wu and Diao (2023)
*Calycina papaeana*	ASL-2022a	OQ079546	OQ079547	Senanayake et al. (2023)
*Calycina parilis*	NN047902	ON993932	–	Wu and Diao (2023)
*Calycina parilis*	352176^(T)^	ON993931	–	Wu and Diao (2023)
*Calycina parvispora*	CBS38594	ON993941	–	Wu and Diao (2023)
*Calycina pseudoaffinis*	CBS 261.75	FR667225	MH872651	Koukol (2011)
*Calycina pseudoaffinis*	CCF 3979^(T)^	NR154761	NG067520	Koukol (2011)
*Calycina riisgaardii*	NN047715	ON993963	OP173649	Wu and Diao (2023)
*Calycina shangrilana*	HKAS 90655a	MK584972	MK591998	Ekanayaka et al. (2019)
*Calycina shangrilana*	HKAS 90655b	MK584971	MK591997	Ekanayaka et al. (2019)
*Calycina subtilis*	PRC 9172	PQ778104	–	Unpublished
*Calycina subtilis*	PRC 9074	PQ778106	–	Unpublished
*Calycina subtilis*	PRC 9038	PQ778105	–	Unpublished
***Calycina xishuangbanica***	**HKAS 139494^(T)^**	**PV138607**	**PV156744**	This study
***Calycina xishuangbanica***	**HKAS 139495**	**PV138608**	**PV156745**	This study
*Chalara africana*	1657a	ON993918	OP173635	Wu and Diao (2023)
*Chalara africana*	16239	ON993919	OP173636	Wu and Diao (2023)
*Chalara bambusicola*	352175^(T)^	ON993923	OP173638	Wu and Diao (2023)
*Chalara cylindrosperma*	CBS65979	ON993927	–	Wu and Diao (2023)
*Chalara longiphora*	16209	ON993936	OP173642	Wu and Diao (2023)
*Chalara longiphora*	77640	ON993937	–	Wu and Diao (2023)
*Chalara pengii*	16010a	ON993942	OP173643	Wu and Diao (2023)
*Chalara pengii*	352179^(T)^	ON993953	OP173646	Wu and Diao (2023)
*Chalara platanicola*	8177	ON993955	–	Wu and Diao (2023)
*Chalara platanicola*	352244^(T)^	ON993956	OP173647	Wu and Diao (2023)
*Chalara qinlingensis*	16647a	ON993959	OP173648	Wu and Diao (2023)
*Chalara qinlingensis*	76918	ON993960	–	Wu and Diao (2023)
*Chlorencoelia torta*	KUS-F52256	JN033400	JN086703	Han et al. (2014)
*Chlorencoelia torta*	ICMP 21732	MH682234	–	Lestari and Chethana (2022)
*Chlorencoelia torta*	JAC14068	MK432798	MK431490	Lestari and Chethana (2022)
*Cylindrocephalum aurea*	1363c	ON993979	OP173654	Wu and Diao (2023)
*Cylindrocephalum aurea*	16031a	ON993982	OP173655	Wu and Diao (2023)
*Cylindrocephalum aureum*	16031b	ON993983	–	Wu and Diao (2023)
*Cylindrocephalum clavatisetosum*	352184^(T)^	ON993984	OP173656	Wu and Diao (2023)
*Cylindrocephalum hughesii*	1612c	ON993994	OP173659	Wu and Diao (2023)
*Cylindrocephalum hughesii*	FMR_12413	KY853434	KY853494	Hernandez-Restrepo et al.(2017)
*Cylindrocephalum kendrickii*	1024c:43800	ON993998	OP173661	Wu and Diao (2023)
*Cylindrocephalum kendrickii*	1024c:3.23367	ON993995	OP173660	Wu and Diao (2023)
*Cylindrocephalum zhejiangense*	16134a	ON993986	–	Wu and Diao (2023)
*Cylindrocephalum zhejiangense*	CGMCC 3.23422^(T)^	ON993985	OP173657	Wu and Diao (2023)
*Gemmina gemmarum*	H.B. 6883	OM456209	–	Lestari and Chethana (2022)
*Gemmina gemmarum*	SBRH 862	KX501127	OM218628	Lestari and Chethana (2022)
*Gemmina* sp.	H.B. 6910	OM456210	OM456211	Lestari and Chethana (2022)
*Laetinaevia epithallina* (*Pezizella epithallina*)	TU39378	KJ559546	KJ559570	Suija et al. (2015)
*Micropeziza umbrinella*	K(M):168087	MZ159422	–	Lestari and Chethana (2022)
*Micropeziza umbrinella*	ZT-Myc-64170	MW489557	–	Lestari and Chethana (2022)
*Microscypha ellisii*	KUS-F52489	JN033418	JN086721	Han et al. (2014)
*Microscypha ellisii*	KUS-F52663	JN033428	JN086731	Han et al. (2014)
*Microscypha* sp.	TNS-F18016	JN033444	JN086745	Han et al. (2014)
*Mollisina uncinata*	TNS-F38901	JN033457	JN086757	Han et al. (2014)
*Mollisina uncinata*	KUS-F52307	JN033404	JN086707	Han et al. (2014)
*Neochalara lolae*	CBS 149065	NR182507	NG149085	Unpublished
*Neochalara spiraeae*	CBS 148332	NR175217	NG081326	Unpublished
*Neochalara spiraeae*	CPC 39565	OK664715	OK663754	Unpublished
*Parachalara olekirkii*	NN043656	ON993965	OP173650	Wu and Diao (2023)
*Parachalara olekirkii*	CGMCC 3.23361^(T)^	NR187035	NG228999	Wu and Diao (2023)
*Psilachnum staphyleae*	KUS-F52105	JN033396	JN086699	Han et al. (2014)
*Rodwayella citrinula*	KUSF52443	JN033414	JN086717	Han et al. (2014)
*Rubropezicula thailandica*	MFLU 16-0592^(T)^	NR_163781	–	Ekanayaka et al. (2019)
*Scleropezicula alnicola*	CBS 200.46	MH856161	MH867686	Vu et al. (2019)
*Scleropezicula alnicola*	CBS 119905	MH863066	–	Vu et al. (2019)
*Zymochalara cyatheae*	CPC:24665^(T)^	NR154509	NG059652	Guatimosim et al. (2016)
*Zymochalara lygodii*	CPC:24699^(T)^	NR154510	NG059653	Guatimosim et al. (2016)

Names in bold indicate the specimens and sequence data from the current study. Names with ^(T)^ indicate type specimens. “–” denotes that sequence data are not available for the respective gene region.

### ﻿Phylogenetic analyses

The newly generated DNA sequences from forward and reverse primers were assembled using BioEdit v.7.2.5 (Hall 1999) to obtain consensus sequences. The concatenated sequences were used to search for closer relatives in the NCBI (Johnson et al. 2008). According to the closely related taxa and recent studies (Lestari and Chethana 2022; Senanayake et al. 2023; Wu and Diao 2023), the newly generated sequences and some published sequences were used for the phylogenetic analyses (Table 1). *Chlorencoelia torta* (KUS-F52256), *C. torta* (ICMP 21732) and *C. torta* (JAC14068) were used as the outgroup taxa in this study, following Lestari and Chethana (2022). The phylogenetic analysis was conducted based on the datasets including reference DNA sequences and newly generated DNA sequences using OFPT (Zeng et al. 2023) with the following protocol. Datasets of each gene region were first independently aligned with the ‘auto’ strategy (based on data size) by MAFFT (Katoh and Standley 2013) and trimmed with the ‘gappyout’ option (based on gaps’ distribution) in TrimAl (Capella-Gutiérrez et al. 2009). The best-fit nucleotide substitution models for each dataset were then selected based on the Bayesian information criterion (BIC) from twenty-two common DNA substitution models with rate heterogeneity by ModelFinder (Kalyaanamoorthy et al. 2017). Afterwards, all datasets were concatenated with partition information for the subsequent phylogenetic analyses. Maximum likelihood with 1000 replicates was performed using ultrafast bootstrap approximation (Hoang et al. 2018) with SH-like approximate likelihood ratio test (SH-aLRT) (Guindon et al. 2010) in IQ-TREE (Nguyen et al. 2015). The consensus tree was summarized based on the extended majority rule. Bayesian inference (BI) analyses were run in the CIPRES Science Gateway v.3.3 (Miller et al. 2010). The best-fit nucleotide substitution models were determined using jModelTest2 on XSEDE (2.1.6). The BI was performed in MrBayes on XSEDE v. 3.2.7a, with four simultaneous Markov Chain Monte Carlo (MCMC) chains and four runs for 3,000,000 generations, with trees sampled at each 300^th^ generations. The first 25% of trees were discarded as burn-in, and BI posterior probabilities (PP) were conducted from the remaining trees. The consensus phylograms were visualized on FigTree v. 1.4.4, and edited with Adobe Photoshop CS6 Extended version 13.0 × 64 (Adobe Systems, USA). Species identifications followed the polyphasic approach recommended by Chethana et al. (2021). The newly generated sequences were deposited at the GenBank, and the accession numbers are given in Table 1.

## ﻿Results

### ﻿Phylogenetic analysis

**Figure 1.**
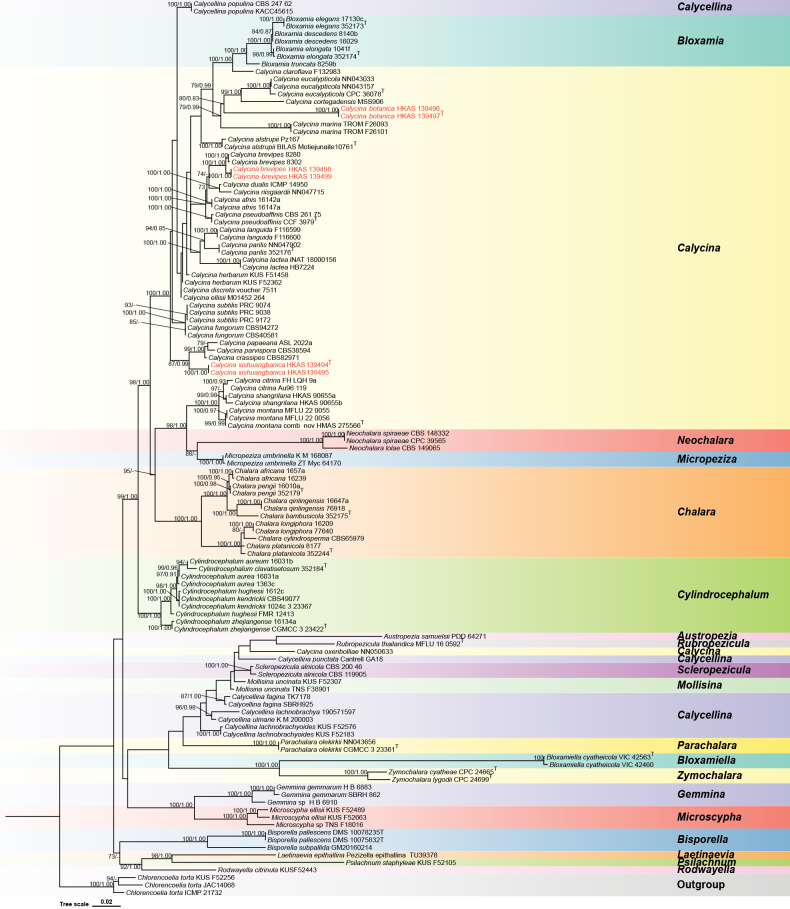
The ML tree is based on the combined LSU and ITS dataset. The MLBP ≥ 70% and BPP ≥ 0.90 are shown at the nodes as MLBP/BPP. The MLBS < 70% and BPP < 0.90 are expressed as a hyphen (“-”). Names with ^T^ indicate type specimens. Names in red indicate newly generated sequences in this study.

The phylogenetic analyses were based on 110 taxa, including *Chlorencoelia torta* isolates (KUS-F52256, ICMP 21732, JAC14068) as the outgroup taxa. The alignment comprised two partitions and 2735 total sites (ITS: 1947 bp; LSU: 788 bp), the matrix had 1072 unique site patterns, with 11.0008% gaps and completely undetermined characters. The topology of the ML tree concurred with the BI tree. The best ML tree with a final optimization likelihood of -17351.016815 is displayed in Fig. 1. Estimated parameters for the tree are as follows: Total tree length (sum of branch lengths): 3.8418; Sum of internal branch lengths: 2.0130 (57.8115% of tree length). *Calycina botanica* clustered with *C. cortegadensis*, with 85% maximum likelihood bootstrap (MLBP) and 0.94 Bayesian posterior probabilities (BPP) support. *Calycina xishuangbanica* clustered with the clade comprising *C. parvispora* (CBS38594), *C. papaeana* (ASL-2022a) and *C. crassipes* (CBS82971), with 99% maximum likelihood bootstrap (MLBP) and 0.99 Bayesian posterior probabilities (BPP) support. *Calycina brevipes* separated from *C. brevipes*, with 100% MLBP and 1.00 BPP support. The phylogenetic result of the current study concurred with previously published studies (Lestari and Chethana 2022, Senanayake et al. 2023).

## ﻿Taxonomy

### ﻿ Calycina botanica

Taxon classificationFungiHelotialesPezizellaceae

L. Luo & K.D. Hyde sp. nov.

DFD51184-46AB-586D-9A83-1DC6E85755C9

Index Fungorum: IF903424

Facesoffungi Number: FoF17337

Fig. 2

#### Etymology.

The epithet *botanica* refers to the collection site “Kunming botanical garden” where the holotype specimen was collected.

**Figure 2.**
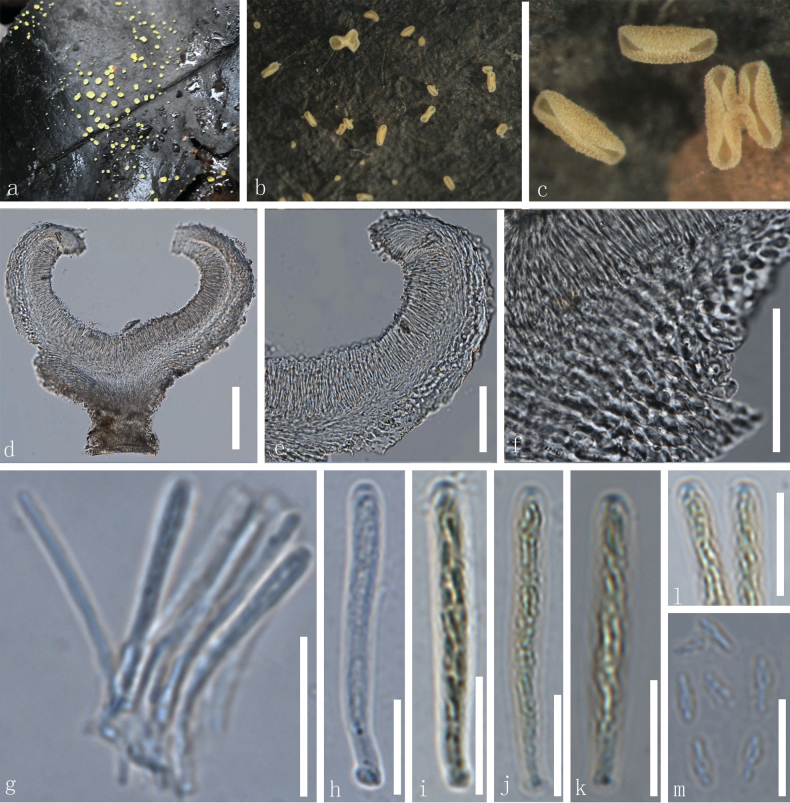
*Calycina botanica* (HKAS 139497, holotype). a–c. Dried ascomata on the host; d. Vertical section of an ascoma; e, f. Excipulum; g. Paraphyses; h–k. Asci (i–k. Asci in Meltzer’s reagent); l. Apices of asci treated with Melzer’s reagent; m. Ascospores. Scale bars: 100 µm (d); 50 µm(e, f); 20µm(g); 10 µm (h–m).

#### Holotype.

HKAS 139497.

#### Description.

***Saprobic*** on dead leaves. ***Sexual morph*: *Apothecia*** superficial, gregarious, 0.2–0.5 mm in diameter, 0.6–1.6 mm high when dry, discoid to cupulate, short stipitate, externally covered with dense tuberculate. ***Disc*** concave, surface smooth, yellow. ***Margin*** flat to involute, yellow. ***Receptacle*** cupulate to discoid, yellow, covered with tuberculate entirely. ***Stipe*** 0.2–0.3 mm in diameter, 0.2–0.4 mm long when dry, cylindrical, solitary, yellow, no hairs. ***Hymenium*** 30–55 µm (x– = 39 µm, n = 42), concave, surface slightly smooth, yellow in fresh and in dry. ***Medullary excipulum*** 13–24 µm (x– = 18 µm, n = 24), thin, hyaline, thin-walled cells of ***textura oblita*** to ***textura porrecta***, 2.3–6.1 µm (x–= 4.5 µm, n = 50) in diameter. ***Ectal excipulum*** 8.5–27 µm (x– = 17 µm, n = 31) thin, thin-walled, smooth, hyaline cells of ***textura globulosa*** to ***oblita***, 1.9–5.7 µm (x– = 3.5 µm, n = 60) in diameter. ***Paraphyses*** 22–34 × 0.8–2.4 µm (x– = 28× 1.7 µm, n = 25), longer than asci, filiform, straight to slightly curved, aseptate, hyaline, smooth, with slightly obtuse apex. ***Asci*** 25–35 × 1.8–3.8 µm (x– = 28.5 × 2.6 µm, n = 50), 8-spored, clavate, straight to slightly curved, inoperculate, hyaline, unitunicate, slightly smooth, with an apical, amyloid pore and rounded ends, J+ in MLZ, tapered short stipitate base. ***Ascospores*** (85/7/2) (3.3–)3.6–5.0(–5.5) × 1.0–2.3 (–2.5) µm, (x– = 4.4 × 1.7 µm, n = 55), partly biseriate, fusoid-clavate with a rounded end and a blunt end, fusiform, 0-1-septate, thin-walled, hyaline, slightly smooth, tapering towards the obtuse ends, one or multi-guttules. ***Asexual morph***: Not observed.

#### Material examined.

• China, Yunnan Province, Kunming City, Kunming Botanical Garden, altitude 1999 m, on the decayed unknown leaf, 15 October 2022, Le Luo, ly1027 (HKAS 139497, holotype); *ibid*., Le Luo, ly1026 (HKAS 139496, paratype).

#### Notes.

*Calycina botanica* (HKAS 139497 and HKAS 139496) clustered the clade comprising *C. cortegadensis* and *C. eucalypticola*, with 85% MLBP and 0.83 BIPP (Fig. 1) statistical supports, and they together formed a clade with *C. marina* (TROM: F26093 and TROM: F26101). *Calycina cortegadensis* was introduced by Crous et al. (2019) from a living twig of *Castanea sativa* from north-western Spain. The new species exhibited morphological differences with *C. cortegadensis* by having yellow apothecia, aseptate paraphyses, and 0–1-septate ascospores whereas the latter species was characterized by brown apothecia, septate paraphyses, aseptate ascospores (Crous et al. 2019). *Calycina botanica* phylogenetically differs from *C. cortegadensis* by 9.3% (46/490 bp) differences in the ITS region and 4.8% (39/815 bp) differences in the LSU region. *Calycina eucalypticola* is only reported in their asexual morphs (Wu and Diao 2023). *Calycina botanica* phylogenetically differs from *C. eucalypticola* by 7.8% (38/490 bp) differences in the ITS region and 5% (41/815 bp) differences in the LSU region.

In addition, our new species shares morphological similarities to *C. marina* in having hyaline, thin-walled cells in the ectal excipulum and smooth, hyaline paraphyses. However, *C. botanica* differs from *C. marina* in having stipitate apothecia, thin-walled, non-gelatinized, *textura oblita* to *porrecta* cells of the medullary excipulum, the tuberculate ornamentation on the receptacle, smaller asci (25–35 µm vs. 58–87 µm), and smaller ascospores (3.6–5.0 µm vs. 8–13 µm), in contrast to the sessile apothecia, thick-walled, gelatinized *textura angularis*-*prismatica* into t*extura intricata*-*porrecta* cells of the medullary excipulum, and the lack of ornamentation on the receptacle of the latter (Baral and Rämä 2015). Based on morphological characters along with the phylogenetic analyses, *C. botanica* is introduced here as a new species.

### ﻿ Calycina xishuangbanica

Taxon classificationFungiHelotialesPezizellaceae

L. Luo & K.D. Hyde sp. nov.

0771CB1B-4C82-5440-85D5-AEA93946412D

Index Fungorum: IF903425

Facesoffungi Number: FoF17338

Fig. 3

#### Etymology.

The epithet refers to the collection site “Xishuangbanna city” where the holotype specimen was collected.

**Figure 3.**
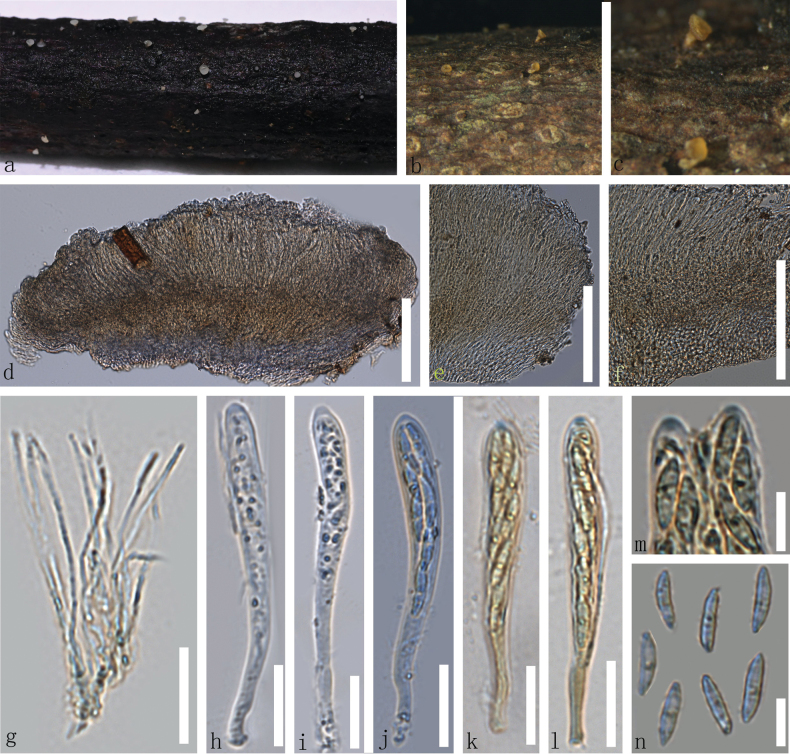
*Calycina xishuangbanica* (**HKAS 139494**, holotype). a–c. Dried ascomata on the wood; d. Vertical section of an ascoma; e, f. Excipulum; g. Paraphyses; h–l. Asci (k, l. Asci in Meltzer’s reagent); m. Apices of asci treated with Melzer’s reagent; n. Ascospores. Scale bars: 100 µm (d–f); 20 µm (g–l); 10 µm (m, n).

#### Holotype.

HKAS**139494**.

#### Description.

***Saprobic*** on dead bark. ***Sexual morph*: *Apothecia*** scattered to partly gregarious, superficial, 0.9–1.3 mm in diameter, 0.3–1.3 mm high when dry, discoid to cupulate, long stipitate, externally no hairs. ***Discs*** concave, surface smooth, white when fresh and yellow when dry. ***Margin*** flat to slightly involute, pale brown to yellow. ***Receptacle*** discoid to cupulate, pale brown to yellow. ***Stipe*** 0.15–0.35 mm in diameter, 0.2–0.8 mm long when dry, cylindrical, solitary, pale brown to yellow. ***Hymenium*** 74–111 µm (x– = 93 µm, n = 35), slightly raised, surface smooth, light brown to light yellow in dry. ***Medullary excipulum*** 20.5–49µm (x– = 36 µm, n = 30), thin, hyaline to light brown, thin-walled cells of ***textura intricata*** to ***porrecta***, 1.6–3.3 µm (x– = 2.5 µm, n = 50) in diameter. ***Ectal excipulum*** 27–63 µm (x– = 44 µm, n =30) thick, thin-walled, smooth, hyaline cells of ***textura porrecta*** to ***textura globulosa***, 1.9–4.8 µm (x = 3.2 µm, n = 60). ***Paraphyses*** 48–83 × 0.8–2.1 µm (x– = 60 × 1.4 µm, n = 35), shorter than asci, filiform, straight, aseptate, hyaline, thin-walled, smooth, with slightly obtuse apex. ***Asci*** 60–90 × 4.6–8.8 µm (x– = 75 × 7.3µm, n = 35), 8-spored, unitunicate, clavate, straight to slightly curved, inoperculate, hyaline, wall apically thickened, laterally relatively thin, slightly smooth, with an apical, amyloid pore and tapered ends, J+ in MLZ. ***Ascospores*** (85/6/2) 9.3–14.5 × 2.9–4.3 µm, (x = 12 × 3.6 µm, n = 86), partially biseriate, filiform, aseptate, thin-walled, hyaline, smooth with tapering towards the obtuse ends, occasionally 1–2 oil guttules, subspherical, hyaline, slightly smooth. ***Asexual morp*h** Not observed.

#### Material examined.

• China, Yunnan Province, Xishuangbanna City, altitude 1787 m, on the decayed unknown twig, 5 September 2022, Le Luo, ly729 (HKAS 139494, holotype); *ibid*., ly751 (HKAS 139495, paratype);

#### Notes.

*Calycina xishuangbanica* (HKAS 139494 and HKAS 139494) clustered with the clade comprising *C. parvispora*, *C. papaeana* and *C. crassipes*, with 99% MLBP and 0.99 BPP support (Fig. 1). *Calycina xishuangbanica* differs from *C. papaeana* in having longer asci (60–90 µm *vs.* 38–58 µm), longer ascospores (9.3–14.5 µm *vs.* 4–9 µm), and pale brown to yellow receptacle and stipe, in contrast to the dark brown to black receptacle and stipe of the latter (Senanayake et al. 2023). *Calycina parvispora* is a saprobe found on dead plant material in Czechoslovakia and New Zealand (Nag Raj and Hughes 1974; Nag Raj and Kendrick 1975). The species was initially introduced by Nag Raj and Hughes (1974) as *Chalara parvispora*, which was later transferred to *Calycina* (Wu and Diao 2023). Similarly, *Calycina crassipes* is a saprobe occurring on dead conifer wood and on the petiole of *Pteridium aquilinum* in Czechoslovakia, New Zealand and the United Kingdom (Nag Raj and Kendrick 1971, 1975; Holubová-Jechová 1984). The species was originally introduced as *Cylindrosporium crassipes* (Ploettnerulaceae, Helotiales) and was later transferred to *Calycina* (Wu and Diao 2023). *Calycina parvispora* and *C. crassipes* are only reported in their asexual morphs (Wu and Diao 2023); *Calycina xishuangbanica* phylogenetically differs from *C. parvispora*, *C. papaeana*, and *C. crassipes* based on the ITS gene region by 4.8%, 7.1%, and 4.1% base pair differences, respectively. Based on the morphological characteristics along with the phylogenetic analyses, *C. xishuangbanica* is introduced here as a new species.

### ﻿ Calycina brevipes

Taxon classificationFungiHelotialesPezizellaceae

(Nag Raj & W.B. Kendr.) W.P. Wu, in Wu and Diao, Fungal Diversity 119: 277 (2023)

2DC1E0EF-0819-526F-93EE-063D815F22F2

Index Fungorum: IF846911

Facesoffungi Number: FoF17339

Fig. 4

#### Holotype.

HKAS 139498.

**Figure 4.**
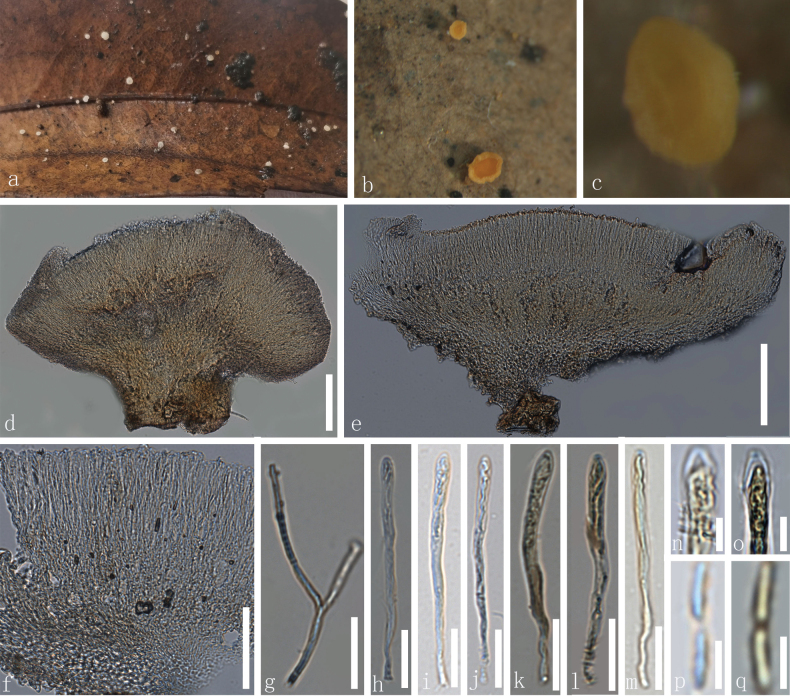
*Calycina brevipes* (HKAS**139498**). a–c. Dried ascomata on the host; d, e. Vertical section of an ascoma; f. Excipulum; g. Paraphyses; h–m. Asci (k–m. Asci in Meltzer’s reagent); n, o. Apical part of ascus in Melzer’s reagent; p, q. Ascospores. Scale bars: 100 µm (d); 50 µm (f); 20 µm(g–m); 5 µm (n–q).

#### Description.

***Saprobic*** on the dead leaf. ***Sexual morph*: *Apothecia*** scattered to partly gregarious, superficial, 1.1–2.4 mm in diameter, 0.9–1.8 mm high when dry, discoid to cupulate, shortly stipitate. ***Disc*** concave, surface slightly rough, cream when fresh and orange when dry. ***Margin*** flat to slightly involute, pale orange to orange. ***Receptacle*** discoid to cupulate, cream yellow to white when fresh and pale orange to orange when dry. ***Stipe*** 0.5–1.2 mm in diameter, 0.6–1.3 mm long when dry, cylindrical, solitary, pale orange to orange. ***Hymenium*** 61–88 µm (x– = 72 µm, n = 30), concave, surface smooth, orange to deeply orange when dry. ***Medullary excipulum*** 22–50 µm (x– = 33 µm, n = 25), thin, hyaline to light yellow, thin-walled, smooth cells of ***textura intricata*** to ***porrecta***, 1.3–2.8 µm (x– =2.0 µm, n = 50) in diameter. ***Ectal excipulum*** 15–34 µm (x– = 24 µm, n =20), thin-walled, smooth, light yellowish cells of ***textura globulosa***, 1.4–4.0 µm (x– = 2.6 µm, n = 60) in diameter. ***Paraphyses*** 48–73 × 1.0–2.5 µm (x– = 64× 1.6 µm, n = 25), longer than asci, filiform, branched, straight to slightly curved, septate, hyaline, thin-walled, rough. ***Asci*** 42–65 × (3.2–) 3.9–5.4(–5.7) µm (x– = 54 × 4.6 µm, n = 42), 8-spored, partially biseriate, clavate, straight to slightly curved, inoperculate, hyaline, unitunicate, apically thin wall, slightly smooth, with an apical, rounded ends, J+ in MLZ. ***Ascospores*** (50/14/2) (4.0–)5.0–7.1(–7.9) × 0.6–1.1(–1.3) µm, (x– = 6.8 × 0.9 µm), fascicled, fusoid-clavate with blunt ends, aseptate, thin-walled, hyaline, obtuse ends, without oil guttules, hyaline, slightly smooth. ***Asexual morph***: See Wu and Diao (2023)

#### Material examined.

• China, Yunnan Province, Puer City, Jingdong County, Ailao Mountain, altitude 2451 m, on the decayed unknown leaf, 23 August 2022, Le Luo, ly423(**HKAS 139498**), ly456(**HKAS 139499**).

#### Notes.

According to the phylogenetic analyses, our collections (HKAS ly423 and HKAS ly456) clustered with the extant *C. brevipes* strains (8280 and 8302) with 100% ML and 1.00 BP support (Fig. 1). *Calycina brevipes* was originally introduced as *Chalara parvispora*, and was later transferred to *Calycina* (Wu and Diao 2023), a saprobic fungus that was reported on the dead leaves of *Podocarpus* and other plants and distributed in Argentina, China and New Zealand (Nag Raj and Kendrick 1975; Gamundí et al. 1978). The anamorph of *C. brevipes* is characterized by the reduced conidiophores, consisting of one basal stalk cell and a terminal phialidic conidiogenous cell, clearly differentiated ellipsoidal venter and cylindrical collarette with a darker lower part, and aseptate, cylindrical conidia (Nag Raj and Kendrick 1975). However, in our collections, no asexual morphs were observed. Despite this, the identification of our collections as the sexual morph of *C. brevipes* is supported by molecular data. The ITS and LSU sequence data of our specimens (HKAS ly423 and HKAS ly456) exhibit 1.1% and 0.8% divergence, respectively, from the known *C. brevipes* strains 8280 and 8302. Given that these levels of genetic variation fall within intraspecific divergence thresholds and that all available strains of *C. brevipes* have only been known in their asexual state, we infer that our collections represent the previously unknown sexual morph of this species. Therefore, the current study presents the first report of the sexual morph of *C. brevipes*, marking its first record from Yunnan Province, China.

## ﻿Discussion

Members of Leotiomycetes exhibit remarkable ecological versatility, inhabiting diverse environments ranging from aquatic to terrestrial ecosystems. They play diverse ecological roles, notably as plant pathogens, endophytes, and saprobes, thereby contributing significantly to nutrient cycling and plant health (O’Brien et al. 2005; Sieber 2007; Baral 2016). In recent years, molecular data have become crucial for the accurate classification of Leotiomycetes taxa (Ekanayaka et al. 2019; Johnston et al. 2019; Quijada et al. 2022). Traditional morphology-based taxonomy alone has often proven insufficient, particularly when resolving phylogenetic relationships among morphologically similar or cryptic species. The integration of multi-gene datasets, particularly ITS, LSU, *RPB*2 and *TEF*1-α, has significantly improved the resolution and reliability of species and generic delimitation (Ekanayaka et al. 2019; Johnston et al. 2019; Quijada et al. 2022). However, in our study, only ITS and LSU were used due to the limited availability of *RPB*2 and *TEF1*-α sequences for closely related taxa in public databases. Molecular phylogenetic analyses have led to the establishment of new genera and the re-definition of existing ones, thereby greatly advancing our understanding of evolutionary relationships within Pezizellaceae and other families in Helotiales. The diversity of Leotiomycetes in China is particularly pronounced, attributable to the country’s heterogeneous climates and diverse ecosystems, which foster a rich array of fungal species within this group (Li et al. 2022, 2024a, b; Phutthacharoen et al. 2022; Su et al. 2022, 2023; Guo et al. 2024; Luo et al. 2024, 2025a, b; Zhang et al. 2024).

The genus *Calycina* exhibits remarkable morphological and ecological diversity, providing valuable insights for fungal taxonomy and systematics. It is well represented in China, with species such as *C. affinis*, *C. brevipes*, *C. eucalypticola*, *C. oxenbolliae*, *C. parilis*, and *C. riisgaardii* reported from provinces including Hubei, Guangxi, Yunnan, and Zhejiang (Wu and Diao 2023). Although 35 *Calycina* species are listed in the Species Fungorum (2025), only 17 species have molecular data, namely *C. afnis*, *C. alstrupii*, *C. citrina*, *C. crassipes*, *C. claroflava*, *C. cortegadensis*, *C. ellisii*, *C. fungorum*, *C. lactea*, *C. languida*, *C. marina*, *C. montana*, *C. oxenbolliae*, *C. papaeana*, *C. parilis*, *C. riisgaardii* and *C. shangrilana*. This highlights the need for further studies incorporating both morphological and molecular approaches to clarify species boundaries within this genus.

Our phylogenetic analyses confirm that the overall topology of the *Calycina* clade aligns with previous studies (Lestari and Chethana 2022; Wu and Diao 2023; Li et al. 2024b), reaffirming its polyphyletic nature. In this study, we describe two new species, *Calycina xishuangbanica* and *Calycina botanica*, from Southwest China. Their placement within *Calycina* is supported by morphological characteristics and multi-gene phylogenetic analyses. Comparisons with morphologically similar species, including *C. papaeana* and *C. cortegadensis*, further justify their recognition as distinct taxa. These findings contribute to the expanding species diversity of *Calycina*.

*Calycina brevipes* (syn. *Chalara brevipes*) was previously known only from its asexual form (Wu and Diao 2023). Although originally placed in *Chalara*, its phialidic, dematiaceous conidial structures remain consistent with *Calycina*. Based on the literature review and phylogenetic analysis, Wu and Diao (2023) supported its transfer to *Calycina*. The species has been reported from Argentina, China, and New Zealand (Nag Raj and Kendrick 1975; Gamundí et al. 1978), yet its sexual morph has not been documented until now. In our study, specimens HKAS ly423 and HKAS ly456 clustered with existing *C. brevipes* strains (8280 and 8302) with strong statistical support (100% ML and 1.00 BP). Despite the absence of an observed asexual morph, the ITS and LSU sequences show only minor divergence (1.1% and 0.8%, respectively) from known *C. brevipes* strains. Given that these genetic differences fall within the intraspecific range and no other *Calycina* species share such close molecular similarity, we confidently assign our collections as the sexual morph of *C. brevipes*.

Our phylogenetic analyses reveal a close relationship between asexual and sexual species within Pezizellaceae. For instance, *Calycina xishuangbanica* clusters with *C. parvispora*, *C. papaeana*, and *C. crassipes*. However, the sexual morphs of *C. parvispora* and *C. crassipes* remain unknown (Wu and Diao 2023), limiting morphological comparisons with *C. xishuangbanica*.

These findings underscore the importance of integrating molecular data to establish connections between sexual and asexual morphs. Many species in *Calycina* are only known from their anamorph, either in nature or in pure culture. Future research should focus on linking sexual and asexual morphs through genetic and morphological analyses to improve the taxonomic framework of this genus. To conclude, our study contributes to this knowledge by reporting the first sexual morph of *C. brevipes* and introducing two new species, *C. xishuangbanica* and *C. botanica*. Moving forward, comprehensive molecular studies will be essential for resolving phylogenetic relationships and further understanding the evolutionary history of this genus.

## Supplementary Material

XML Treatment for Calycina botanica

XML Treatment for Calycina xishuangbanica

XML Treatment for Calycina brevipes

## ﻿Additional information

### Use of AI

No use of AI was reported.

### Author ORCIDs

Le Luo https://orcid.org/0009-0006-7213-0498

Kandawatte Wedaralalage Thilini Chethana https://orcid.org/0000-0002-5816-9269

Qi Zhao https://orcid.org/0000-0001-8169-0573

Vinodhini Thiyagaraja https://orcid.org/0000-0002-8091-4579

Kitiphong Khongphinitbunjong https://orcid.org/0000-0002-4548-2790

Fatimah Al-Otibi https://orcid.org/0000-0003-3629-5755

Kevin D. Hyde https://orcid.org/0000-0002-2191-0762

### Data availability

All of the data that support the findings of this study are available in the main text.
